# The effect of therapeutic bronchoalveolar lavage in combination with glucocorticoids on children with acute exogenous lipoid pneumonia

**DOI:** 10.1111/crj.13575

**Published:** 2023-02-19

**Authors:** Sen Yang, Shangzhi Wu, Jiaxing Xu, Yuneng Lin, Zhanhang Huang, Xiaowen Chen, Qingyun Xu, Dehui Chen, Chengyu Lu

**Affiliations:** ^1^ Department of Pediatrics First Affiliated Hospital of Guangzhou Medical University Guangzhou Guangdong China

**Keywords:** bronchoalveolar lavage, children, exogenous lipoid pneumonia, glucocorticoids therapy

## Abstract

**Background:**

Exogenous lipoid pneumonia (ELP) is a rare disease caused by the inhalation of oily materials in the alveoli with the pathological characterization by the presence of laden‐lipid macrophages in the respiratory specimens. At present, the treatment norm for ELP has not well defined, and so the aim of this study is to evaluate the effect of bronchoalveolar lavage in combination with glucocorticoids on children with ELP.

**Methods and Materials:**

We retrospectively reviewed 17 children with a confirmed history of exogenous oily materials aspiration, admitted to the First Affiliated Hospital of Guangzhou Medical University from June 2012 to December 2021. Clinical features, blood investigations, tomographic evaluations, therapeutic bronchoalveolar lavage and glucocorticoids use were carried out at the beginning of therapy and throughout a follow‐up period.

**Results:**

The included children are the median age of 2 years. Fever, dypnea and tachypnea were the most common symptoms. The most common radiological features were airspace consolidations (15, 93.75%). Chest CT scans showed areas of consolidation with air bronchogram (15, 93.75%), poorly defined centrilobular nodules (13, 81.25%), areas of ground‐glass attenuation (11, 68.75%) and ‘crazy‐paving’ pattern (6, 37.5%) in the both lower, right middle lung lobes. Neutrophil percentage of peripheral blood and bronchoalveolar lavage fluid exhibited a significantly higher than the normal range. After treatment with multiple bronchoalveolar lavages and local administration of budesonide during the hospital stay, taken by oral prednisolone (1 ~ 2 mg/kg) after discharge, all of children became asymptomatic and presented normal radiological imagings in the follow‐up period.

**Conclusion:**

The most frequently findings in the CT scan of ELP were consolidations and ground‐glass attenuation in the both lower and right middle lung lobes. Multiple bronchoalveolar lavages in combination with oral prednisolone for children who had a confirmed history of exogenous oily substances ingestion were an efficient and safe for the clearance of oily materials from the lung and the prevention of fibrosis. This strategy contributed to reducing the damage of ELP in children patients.

## Graphical abstract

The most common radiological features were airspace consolidations in children with acute exogenous lipoid pneumonia. Bronchoalveolar lavage with glucocorticoids was an efficient and safe for with acute exogenous lipoid pneumonia.

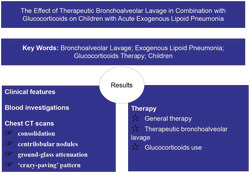

## 1 INTRODUCTION

Lipoid pneumonia (LP) is a rare disease caused by inhalation or aspiration of oily substances and has been reported with an incidence of only 1% ~ 2.5% in autopsy series.^1^ Based on the source of the lipid, LP is categorized as follows: exogenous lipoid pneumonia (ELP), due to an episode of aspiration of a large quantity of an oil‐based product, and endogenous LP, related to uncommon medical conditions.^2^, ^3^ Historically, Yeung et al.^4^ reported the first cases of exogenous lipoid pneumonia in one adult and in four children after chronic exposure to mineral oil nose drops and laxatives. Since then, most cases have been reported in patients with a predisposition to aspiration, such as mental retardation, cleft palate, anatomic or functional abnormality in swallowing.^4^, ^5^, ^6^, ^7^, ^8^ But there have been some reports of exogenous LP caused by traditional folk remedies.^9^ Various causative agents can cause LP, depending on local life style and cultural differences among patients.^3^, ^10^, ^11^ In China, kerosene, as a special kind of mineral oil for worship, has been reported as a common cause of exogenous LP.^3^, ^10^

Due to lack of specific radiological features and manifestations of LP and awareness about this disease, diagnosis can be missed or delayed at the right time, which may lead to progress and unnecessary therapy.^2^, ^12^ The nature history and outcome of LP are greatly variable mainly depending on the type, quantity, chemical properties and distribution of inhaled materials and are related to pulmonary tissue injury degree.^13^ At present, the treatment strategy for LP is not well defined in the literature and guidelines, but there is a consensus that the main measure is primarily supportive and generally conservative and ceases to exposure to the lipid substances. In 2020, a systematic review indicated that therapeutic lung lavage might be effective and safe treatment with long‐term benefits for exogenous LP.^12^ In children patients with diffuse pulmonary lesion, aggressive therapy has been reported.^7^ However, therapy measures about the LP derives largely from case reports and case series, not long‐term observational studies.^12^ In this study, we evaluated the radiological and clinical features of children LP patients used by therapeutic segmental bronchoalveolar lavage in combination with prednisolone.

## 2 METHODS AND MATERIALS

The study was approved by the institutional ethic review committee, which waived the requirement for written informed consent because of the nature of the retrospective study.

### 2.1 Study design

All inpatient children under 18 years of age with an admission diagnosis of ELP and a confirmed history of exogenous oily materials aspiration were retrospectively reviewed from June 2012 to December 2021 from the medical records at First Affiliated Hospital of Guangzhou Medical University, a tertiary care, free‐standing public hospital. Medical records were retrospectively reviewed by using a standardized form to collect demographic data, clinical manifestations, laboratory test results and radiological findings. All patients with established ELP were evaluated by the senior attending paediatrician at the inpatient units on the basis of signs, symptoms and laboratory/imaging findings during hospital stay. The exclusion criterion was suspected with a history of or no evidence of exogenous substances inhalation. A total of 17 children patients was included in the final analysis. CT scans of all children found diffuse pulmonary lesion at admission. Supporative measures, such as oxygen therapy, empirical antibiotic therapy, local or systematic corticosteriods use to slow the inflammatory response, were performed in children with clinical symptoms or severe lung damage. The number of bronchoscopic lung lavage was carried out in terms of the severity of clinical symptoms/signs and radiological findings.

### 2.2 Data collection

We collected data on demographics (sex, age and weight), symptoms/signs, hospital stay, blood investigations (whole blood count, neutrophil/lymphocyte/monocyte percentage, C reactive protein, procalcitonin, arterial blood gas and peripheral lymphocyte subset percentage), radiological imagings, bronchoalveolar lavage fluid analysis (lymphocyte subset percentage and neutrophil/lymphocyte/macrophage percentage), source of inhaled oil substance, comorbidity, number of therapeutic bronchoalveolar lavage and clinical course. Clinical course was classed into four categories as follows: complete or partial resolution; no charge; progression and lost to follow‐up. Bronchoscopic data on lymphocyte subset was determined by flow cytometry.

### 2.3 Radiological imaging's evaluation

**FIGURE 1:**
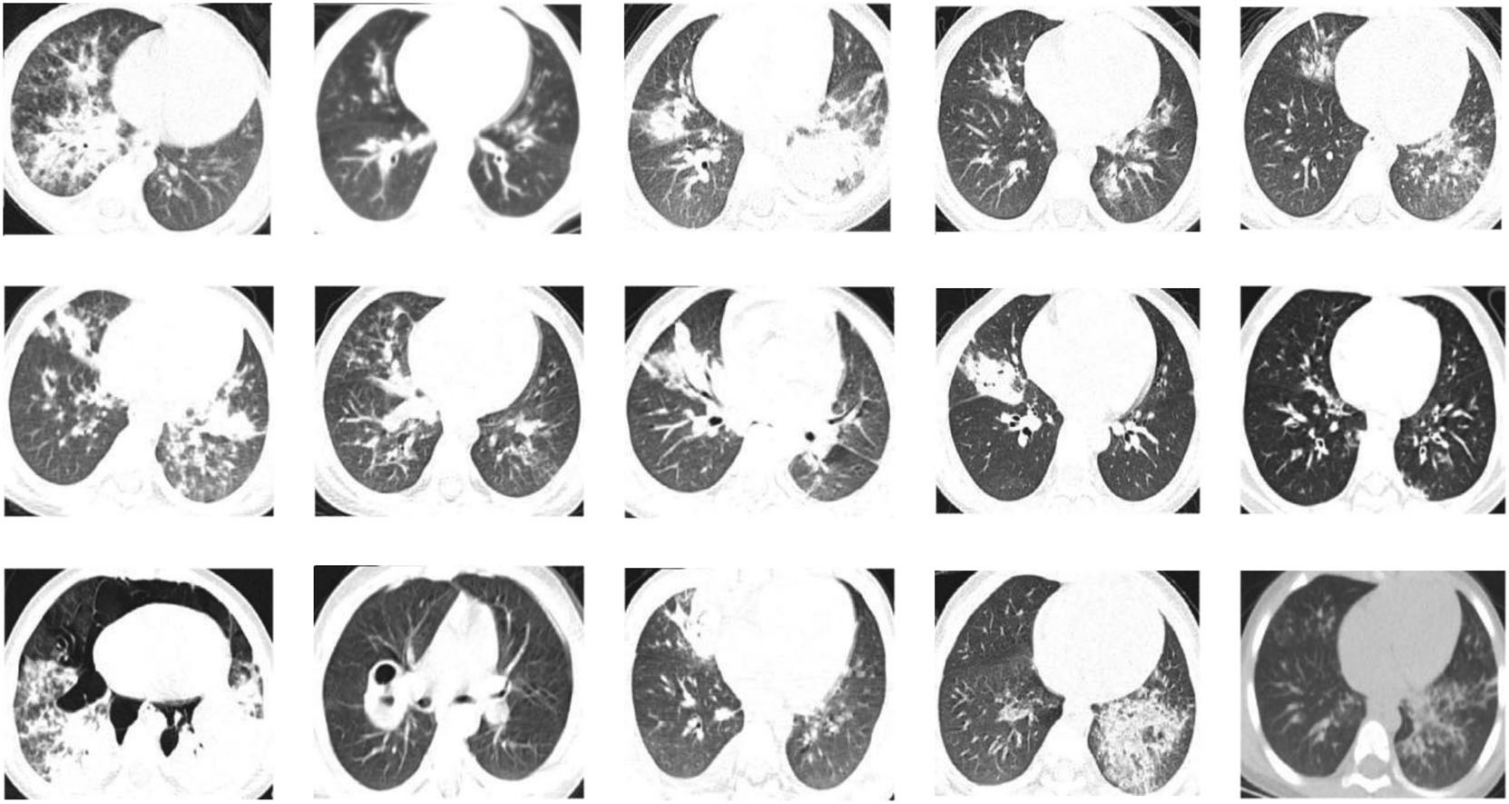
CT findings of 15 patients before admission, in two of which were not available

High‐resolution CT (HRCT) scans of all children were evaluated for the presence and distribution of the following findings: air‐space consolidation, ground glass attenuation, crazy‐paving pattern, and poorly defined centrilobular nodules.^14^, ^15^, ^16^, ^17^ Two investigators independently reviewed the HRCT images, and discrepancies on these findings were discussed by consensus. Criteria for these findings were checked in each patient (Figure 1). Crazy‐paving pattern was defined as patchy well‐defined areas of ground‐glass opacity with superimposed septal thickening.

### 2.4 Statistical analysis

Data were expressed as medians with interquartile ranges for continuous variables and as numbers with percentages for categorical variables. Among two groups, continuous and categorical variables were compared by using the Mann–Whitney U test and χ^2^ test or Fisher's exact test, respectively. *P* ≤ 0.05 was considered statistically significant. Statistical analysis was carried out by using IBM SPSS Statistics for Windows, verision 25.0 (IBM Corp., Armonk, New York) software.

## 3 RESULTS

### 3.1 Manifestations and radiological features of the children LP patients

In our study, a total of 17 patients were included, with median age of 2 years (Interquartile Range, IQR [1.48 ~ 4.23]), in two of which had severe malnutrition and in one of which had obesity, the remaining 14 patients had no risk factors. The most frequent symptoms were cough (*n* = 14), fever (*n* = 14) and tachypnea (*n* = 7). Clinical visit data were available for the 17 children who finally were asymptomatic and presented normal radiological imagings. One patient taking sewing machine oil for 2 days had a severe pneumothorax but a good clinical evolution. Corticosteroid and preventive antibiotics use were administered in 12 of the 17 children. Bronchoscopic lavage using normal saline was performed in all children. Characteristics of the patients are listed in the table 1.

**TABLE 1:** Baseline characteristics of the patients

Feature	Values (*n*, % or median [IQR])
Sex (male/female)	17 (9/8)
Age, years	2 (1.48 ~ 4.23)
Weight (kg)	11.5 (10.0 ~ 14.5)
Comorbidity	
Obesity	1
Severe malnutrition	2
Hospital stay, days	10 (7.75 ~ 19)
Source of inhaled oil	
Mineral oil	12
Vegetable oil	1
Other	4
Duration of symptom, days	
Fever	6 (4 ~ 13.25)
Cough	11 (9 ~ 22)
Dyspnea	5 (4 ~ 6.75)
Chest pain	6.5 (5.75 ~ 7.25)
Vomit	3 (1 ~ 3.25)
Corticosteroid treatment	
Intravenous or oral	12 (70.59)
Aerosol inhalation	10 (58.82)
Number of bronchoalveolar lavage	2 (1 ~ 2)
Course of disease before admission, days	3 (1 ~ 5)
Clinical course	
Complete or partial resolution	17
No change	0
Progression	0
No follow‐up	0

Laboratory findings, such as arterial blood gas analysis (ABGA), peripheral blood investigations, bronchoscopic lavage fluid (BALF) analysis and CT features are presented in Figure 1 and Table 2. Mild hypoxemia and hypocapnia were noted. BALF analysis was found in 8 of 17 patients who underwent bronchoscopy, and the increased neutrophilic granulocyte percentage was observed in BALF analysis and peripheral blood investigations (Table 2).

**TABLE 2:** Laboratory and computed tomography findings of the patients (*n*, % or median [IQR])

Parameters	Values
PaO2, mm Hg^a^	87.3 (82.4 ~ 102.2)
PaCO2, mm Hg^a^	32.8 (29.6 ~ 35.6)
Peripheral Blood investigation	
WBC, *10^9^/L	15.63 (10.53 ~ 20.25)
N, %	64.1 (45.85 ~ 71.6)
L, %	25.9 (20.8 ~ 43.25)
M, %	6.6 (4.9 ~ 10.25)
Inflammatory indicator	
CRP	6.225 (1.55 ~ 7.86)
PCT	0.3 (0.21 ~ 1.24)
Peripheral lymphocyte subset investigation	
CD3^+^T, %	67.7 (61.6 ~ 68.7)
CD3^+^CD4^+^Th, %	35 (30.3 ~ 37.6)
CD3^+^CD8^+^Ts, %	23.9 (21.7 ~ 29)
Th/Ts, %	1.14 (1.04 ~ 1.46)
CD3^−^CD19^+^B, %	20.6 (20.3 ~ 25.5)
CD3^−^CD16^+^CD56^+^NK, %	7.4 (7.1 ~ 9.1)
Bronchoalveolar lavage fluid (BALF) analysis	
CD3T%	76.9 (73.95 ~ 83)
CD3/CD4(Th) %	20.1 (16.7 ~ 25.5)
CD3/CD8(Ts) %	43.4 (38.15 ~ 56.9)
Th/Ts	0.46 (0.24 ~ 0.67)
*N*, %	64 (28.13 ~ 84.25)
Mθ, %	13.165 (5.58 ~ 61.75)
E, %	0.125 (0 ~ 0.44)
L, %	2.415 (1.71 ~ 5.88)
Computed tomography	
Location	
Right upper lobe	1 (6.25%)
Right middle lobe	12 (75%)
Right lower lobe	12 (75%)
Left upper lobe	4 (25%)
Left lower lobe	12 (75%)
Findings	
Ground‐glass opacity	11 (68.75%)
Consolidation	15 (93.75%)
Poorly defined centrilobular nodules	13 (81.25%)
Crazy‐paving pattern	6 (37.5%)

Abbreviations: WBC, white blood count; N, neutrophile; L, lymphocyte; M, monocyte; CRP, C reaction protein; PCT, procalcitonin; Ts, T suppressor; Th, T helper; NK, natural killer; Mθ, macrophage; E, eosinophilic.

^a^ The PaO2 and PaCO2 measurements were obtained with the patients breathing room air.

In most cases, the lesions were multilobular; both lungs were equally involved; the right lung was more extensively involved than the left lung. On CT scan, the most common radiological features in the patients with ELP were bilateral areas of air‐space consolidation (15, 93.75%) and poorly defined centrilobular nodules (13, 81.25%). The children had no pleural effusion, lymph‐node enlargement or any other chest abnormality. The presence of ground‐glass attenuation was observed in 11 cases and ‘crazy‐paving’ pattern (septal thickening) in 6 cases (Figure 1).

### 3.2 Comparison of two groups from different source of inhaled oily materials

On the basis of aspirated different oily substances, the 17 patients were classed into two groups: mineral oil group and other group. Of those, lamp kerosene was inhaled in nine cases, machine oil two cases, white gas one case, butter one case, liquid for killing mosquito two cases, banana oil one case and glue one case. The mineral oil group had a significantly higher white blood count than the other group but no statistical significance. In comparison with the mineral oil group, the other group demonstrated BAL lymphocytosis. The origin of oily materials was no obviously affected in prognosis and laboratory findings of ELP (Table 3).

**TABLE 3:** Comparison of clinic‐radiological features from the source of mineral oil and other

	Mineral oil	Other	*P*
Sex (male/female)	12 (6/6)	5 (3/2)	0.707
Age (years)	1.80 (1.58 ~ 4.23)	2.2 (1.3 ~ 2.5)	0.712
Weight, kg	11.9 (9.9 ~ 15.1)	10.9 (10 ~ 14)	0.916
Hospital stay, days	11 (7.25 ~ 22.5)	9 (8 ~ 15)	0.721
Course of disease before admission, days	3 (1 ~ 4.75)	3 (1 ~ 5)	0.956
Number of bronchoalveolar lavage	1.5 (1 ~ 2.25)	1 (1 ~ 2)	0.734
Peripheral Blood investigation			
WBC, *10^9^/L	17.1 (10.75 ~ 20.25)	10.25 (8.85 ~ 15.1)	0.433
*N*, %	0.643 (0.48 ~ 0.72)	0.49 (0.41 ~ 0.65)	0.296
L, %	0.249 (0.21 ~ 0.42)	0.3885 (0.28 ~ 0.44)	0.433
M, %	0.073 (0.05 ~ 0.1)	0.0615 (0.06 ~ 0.08)	0.948
Inflammatory indicator			
CRP	6.225 (1.88 ~ 7.8)	4.475 (2.01 ~ 8.17)	0.671
PCT	0.29 (0.2 ~ 2.06)	0.3 (0.24 ~ 1.11)	>0.999
Peripheral lymphocyte investigation			
CD3^+^T, %	67.7 (64.65 ~ 68.2)	61.65 (54.33 ~ 68.98)	>0.999
CD3^+^CD4^+^Th, %	35 (32.65 ~ 37.15)	28.6 (24.1 ~ 33.1)	0.564
CD3^+^CD8^+^Ts, %	23.9 (22.8 ~ 26.45)	26.55 (23.33 ~ 29.78)	>0.999
Th/Ts, %	1.46 (1.25 ~ 1.64)	1.06 (1.02 ~ 1.1)	0.248
CD3^−^CD19^+^B, %	25.5 (23.05 ~ 28)	16.4 (14.45 ~ 18.35)	0.083
CD3^−^CD16^+^CD56^+^NK, %	7.1 (5.55 ~ 8.1)	19.65 (13.53 ~ 25.78)	0.248
Bronchoalveolar lavage fluid analysis			
CD3T %	76.05 (74.58 ~ 76.98)	88.8 (80.65 ~ 90.2)	0.480
CD3/CD4(Th) %	14 (10.3 ~ 16.7)	25.5 (21.38 ~ 35.03)	0.034
CD3/CD8(Ts) %	38.15 (32.1 ~ 39.58)	60.2 (56.9 ~ 62.75)	0.034
Th/Ts	0.67 (0.54 ~ 1.49)	0.11 (0.07 ~ 0.24)	0.034
*N*, %	77 (51 ~ 86.67)	20	
Mθ, %	10.33 (4 ~ 16)	78.25	
E, %	0 (0 ~ 0.5)	0.25	
L, %	2.5 (2.33 ~ 7)	1.5	
Computed tomography			
Location			0.025
Right upper lobe	0 (0%)	1 (20%)	
Right middle lobe	9 (75%)	2 (40%)	
Right lower lobe	10 (83.33%)	2 (40%)	
Left upper lobe	2 (16.66%)	2 (40%)	
Left lower lobe	9 (75%)	1 (20%)	
Findings			0.776
Ground‐glass opacity	8 (66.67%)	3 (60%)	
Consolidation	12 (100%)	3 (60%)	
Poorly defined centrilobular nodules	11 (91.66%)	2 (40%)	
Crazy‐paving pattern	4 (33.33%)	2 (40%)	

## 4 DISCUSSION

Exogenous lipoid pneumonia (ELP) is a rare condition that can be difficult to recognize and results from accumulation of oily materials in the alveoli that are aspirated from vegetable, mineral and animal origins, characterized by lipid‐laden macrophages in the sputum or bronchoscopy lavage.^5^, ^18^, ^19^ In the present study, we demonstrated that acute ELP frequently is symptomatic, with respiratory symptoms reported by almost all of affected children patients who had a median 2 years of age. Bilateral areas of consolidative and ground‐glass opacities were the most common radiological findings, but crazy‐paving pattern on CT scan images was present in less than one‐half of affected individuals. Aspirated mineral oil was the most commonly implicated materials. After discontinuation of the causative agent and supportive treatment, such as oxygen therapy, bronchoalveolar lavage and glucocorticoid use, a large percentage of the children patients significantly improved clinically and radiologically. All of the patients had acute ELP in our study rather than chronic. Pathologically, acute ELP is generally believed to be caused by a foreign body response to fatty substances in the lung lobe^20^; therefore, we found that all affected individuals had a markedly higher percentage of neutrophils in BAL fluid and peripheral blood in comparison with the normal range. Acute ELP is more common in the literature report and occurs in association with large‐dose inhalation or aspiration in specific clinical settings, such as accidental ingestions in children or fire‐eaters, suicide attempts and illegal drug use.^1^, ^9^, ^13^

To precisely review the effect of bronchoalveolar lavage on acute ELP, we only included patients with a confirmed history of aspiration, clinical symptoms and CT scan images.^13^ However, due to lack of specific radiological and clinical features and no awareness of LP, LP is difficult to establish and sometime histopathologic confirmation of the diagnosis may be necessary from respiratory specimens.^21^ Although many of the reports have used lipoid‐laden macrophage in the sputum or BAL specimen as a diagnostic marker for LP, its specificity and accuracy have been questioned by some researchers.^21^ In the present study, BAL fluid analysis shows the presence of lipoid‐laden macrophage in the only one case. Therefore, diagnosis of ELP should be based on the triad of history of mineral oil inhalation, compatible radiological imagings and presence of intra‐alveolar lipids and/lipid‐laden macrophages.^4^, ^6^, ^18^, ^20^

BAL is a successful method recommended in the therapy of pulmonary alveolar proteinosis,^22^, ^23^, ^24^ but a great many investigations and case‐reports have demonstrated good response of whole/segmental lung lavage in the therapy of individuals with LP. Whole or segmental BAL is a simple and safe procedure that does not require general anaesthesia but cough and hypoxemia may eventually occur during the procedure.^24^ BAL may result in transient hypoxemia in few children individuals, but it was swiftly corrected by nasal oxygen, and children caregivers did not report any complication after the procedure.^25^ In our research, all affected children were performed by BAL and had full resolution until the last follow‐up, even multiple BAL in some patients on the basis of the clinical‐radiological patterns of paediatric ELP.^23^ Indeed, it has been reported in the literature that 6 months after ELP diagnosis, untreated children had recurrent respiratory infections that required antibiotic therapy and chest CT pattern still showed areas of ground‐glass opacities in the lower lobes and atelectasis.^11^ BAL is a good strategy recommended in the treatment of paediatric acute ELP in terms of our clinical research.^25^, ^26^

Regardless of location, the inflammatory response can destroy the alveolar walls and the interstitium, and the resultant fibrosis can occasionally progress to end‐stage lung disease.^18^, ^20^, ^27^, ^28^ In this study, we divided all patients into two groups based on the source of aspirated substances; the clinical‐radiological features were found no statistical difference in both group.

There are also several limitations in the present study. First, this was a respective study conducted in only one hospital so that many confounding factors and various bias could not be avoided due to the inherent nature of the retrospective study. Second, the radiological findings of all children varied greatly from CT scan images at initial presentation to X‐ray films during the follow‐up period; therefore, we did not get quantitative data to compare. Finally, the number of children was insufficient to reach a credible and robust conclusion. In the future, larger multicenter prospective cohorts are needed to elucidate the risks and benefits of bronchoscopic lavage and glucocorticoids on children with ELP.

In conclusion, diagnosis of acute ELP is often difficult. Causative agents of ELP can vary depending on differences in culture and life style of the affected countries and regions. Diagnosis of acute ELP should be based on confirmed exposure to lipid substances and clinical‐radiological features. Sufficient exposure data are necessary to reach a diagnosis of acute ELP and to differentiate it from pneumoconiosis.

## CONFLICT OF INTEREST

All of authors declare that they have no conflict of interest.

## AUTHOR CONTRIBUTIONS

SY and SZW were involved in collecting the medical records and drafted the first manuscript. JXX and YNL were engaged in data extraction and reviewing the quality of included studies with the aid of ZHH, XWC and QYX. Statistical analysis was carried by YNL and ZHH. All authors reviewed this article and contributed the revisions. DHC and CYL are the guarantor for this article.

## ETHICS STATEMENT

The study was approved by the institutional ethic review committee, which waived the requirement for written informed consent because of the nature of the retrospective study.

## Data Availability

The data that support the findings of this study are available from the corresponding author upon reasonable request.
